# Mapping the local viscosity of non-Newtonian fluids flowing through disordered porous structures

**DOI:** 10.1038/s41598-020-68545-7

**Published:** 2020-07-16

**Authors:** U. Eberhard, H. J. Seybold, E. Secchi, J. Jiménez-Martínez, P. A. Rühs, A. Ofner, J. S. Andrade, M. Holzner

**Affiliations:** 1 Department of Civil, Environmental and Geomatic Engineering, ETH Zurich, 8093 Zurich, Switzerland; 2 Department of Environmental Systems Science, ETH Zurich, 8092 Zurich, Switzerland; 3 Department of Materials, ETH Zurich, 8093 Zurich, Switzerland; 4 Swiss Federal Institute of Aquatic Science and Technology, EAWAG, 8600 Dübendorf, Switzerland; 5 Swiss Federal Institute for Forest, Snow and Landscape Research, WSL, 8903 Birmensdorf, Switzerland; 6 Departamento de Física, Universidade Federal do Ceará, 60451-970 Fortaleza, Ceará Brazil

**Keywords:** Engineering, Soft materials

## Abstract

Flow of non-Newtonian fluids through topologically complex structures is ubiquitous in most biological, industrial and environmental settings. The interplay between local hydrodynamics and the fluid’s constitutive law determines the distribution of flow paths. Consequently the spatial heterogeneity of the viscous resistance controls mass and solute transport from the micron to the meter scale. Examples range from oil recovery and groundwater engineering to drug delivery, filters and catalysts. Here we present a new methodology to map the spatial variation of the local viscosity of a non-Newtonian fluid flowing through a complex pore geometry. We use high resolution image velocimetry to determine local shear rates. Knowing the local shear rate in combination with a separate measurement of the fluid’s constitutive law allows to quantitatively map the local viscosity at the pore scale. Our experimental results—which closely match with three-dimensional numerical simulations—demonstrate that the exponential decay of the longitudinal velocity distributions, previously observed for Newtonian fluids, is a function of the spatial heterogeneity of the local viscosity. This work sheds light on the relationship between hydraulic properties and the viscosity at the pore scale, which is of fundamental importance for predicting transport properties, mixing, and chemical reactions in many porous systems.

## Introduction

Many industrial processes exploit artificial porous structures to separate particles in filters^1^, enhance chemical reactions in catalysts^2^, improve mass and heat transfer in packed bed reactors^3^ or to optimize transport in chromatographic devices^4,5^. In groundwater remediation^6^ or oil recovery^7^, the natural porous structure controls the efficiency of the extraction process. Disordered porous structures are also frequently found in biological and medical systems, affecting processes such as transport of mucus^8^ or blood flow through the kidney and other tissues^9–11^. Often the fluids involved in these processes are so-called non-Newtonian fluids showing a complex stress–strain relation due to the presence of additives^12,13^. Consequently, their flow resistance cannot be described by a single viscosity constant, as in the case of Newtonian fluids. Instead, the local viscosity is a nonlinear function of the shear rate and in some cases even a function of time^14^. In heterogeneous pore structures flows exhibit a wide range of velocities and different shear rates. Consequently, the local viscosity of non-Newtonian fluids is also spatially variable. This variability in the local hydraulic resistance impacts the distribution of local flow velocities through the interstitial pore space, ultimately affecting transport, and mixing processes^15^. While the measurement of the bulk viscosity of non-Newtonian fluids with rheometers and microfluidic platforms is rather common^16–19^, the mapping of the local viscosity at the pore scale in a complex geometry is challenging. The reason for this is that the fluid’s constitutive behavior has to be carefully matched with the experimental operating condition in order to achieve reliable measurements in the nonlinear consititutive regime. Therefore, the study of non-Newtonian flows has mainly been addressed computationally^20–23^ or using simple geometries^24^.

Here we present a microfluidic method to quantitatively map the heterogeneity of the local viscosity of a non-Newtonian single-phase flow through a complex quasi-two-dimensional pore structure. The key innovation of our approach relies on the combination of high-resolution image velocimetry with a seperate constitutive characterization of the non-Newtonian fluid over a broad range of shear rates. By combining the rheology measurement with the shear rates calculated from the velocimetry measurements, we are able to determine the local viscosity of the fluid at the pore scale. In order to benchmark our method, we compare the experimental results with extensive three-dimensional numerical simulations^25^, where we solve the equations of motion of a non-Newtonian fluid with the same constitutive law, the same flow parameters and pore geometry as in the experiment. The good agreement between these theoretical predictions and the experimental results shows that our approach allows to quantitatively map the variations of the local viscosity field. Moreover it shows that all relevant effects have been considered sufficiently in our analysis and that our rheology measurement correctly describes the constitutive response of our fluid. Moreover, we find that the exponential decaying flow velocity distributions, formerly observed for Newtonian flows^26,27^, becomes wider the more heterogeneous the distribution of the viscosity in the interstitial pore space is. Our analysis opens a new perspective to exploit the flow of non-Newtonian fluids through disordered porous structures, by predicting local changes in viscous and hydraulic resistance and ultimately controlling mass and solute transport.

### The equations of motion of non-Newtonian flows

The flow of an incompressible fluid through the interstitial pore space under isothermal steady-state conditions can be described by mass and momentum conservation as

1 $$\begin{aligned} \nabla \cdot \vec {u}=\, & {} 0 \end{aligned}$$

2 $$\begin{aligned} \rho (\vec {u}\cdot \nabla \vec {u})= & {} -\nabla p+\nabla \cdot {\mathsf {T}}, \end{aligned}$$

including appropriate boundary conditions^28^. Here $\rho$ is the fluid’s density and $\vec {u}$ and *p* are its velocity and pressure, respectively. The variable ${\mathsf {T}}$ is the deviatoric stress tensor, which depends on the fluid’s constitutive law^28^. In the case of a Newtonian fluid it follows a simple linear relation ${\mathsf {T}}=2\mu {\mathsf {E}}$, where the variable

3 $$\begin{aligned} {\mathsf {E}}=\frac{1}{2}\left( \nabla \vec {u} +(\nabla \vec {u})^T\right) \end{aligned}$$

is the shear-strain-rate tensor^28^, with its components running over the three spatial coordinates *x*, *y*, and *z*. For most non-Newtonian fluids, the classical Newtonian relation between stress and deformation can be generalized as

4 $$\begin{aligned} {\mathsf {T}}=2\mu ({\dot{\gamma }}){\mathsf {E}}, \end{aligned}$$

where the local viscosity $\mu ({\dot{\gamma }})$—from now on simply called viscosity—is a nonlinear function of the second principal invariant ${\dot{\gamma }}$ of the shear-strain-rate tensor alone^28^. This variable ${\dot{\gamma }}$ is defined by

5 $$\begin{aligned} {\dot{\gamma }}=\sqrt{2{\mathsf {E}}:{\mathsf {E}}}, \end{aligned}$$

and describes the scalar shear-rate in the case of simple shear flow^28^. Several empirical parametrizations for the relation between $\mu$ and ${\dot{\gamma }}$ have been proposed, where the most common ones are the power-law model, the Cross model, the Herschel-Buckley model, and the Carreau model, only to mention a few^29,30^. Among these models the last one has widely been used due to its ability to describe the rheology of a wide range of relevant solutions^31^. Given a viscosity-shear relation $\mu ({\dot{\gamma }})$ and a velocity field $\vec {u}(x,y,z)$, the incompressible flow problem, described by the solutions of Eqs. (1) and  (2), is fully characterized, and the local viscosity can be readily calculated using Eqs. (3) and (5).

## Experimental results

### Measurement of the velocity field and shear rates

In order to determine the local viscosity field within the interstitial pore space of a microfluidic device, the flow field has to be measured with a high spatial resolution. This is particularly relevant for calculating local shear rates from the experimentally measured velocities based on finite differences ("Methods" section). We accomplish this task with an advanced optical velocimetry technique, called Ghost Particle Velocimetry (GPV)^32,33^. GPV yields flow velocities using sub-resolution tracers similar to micro Particle Image Velocimetry^34^ ($\mu \text {PIV}$). The tracer particles are added in low concentration to the sample fluid in order to avoid aggregations^35^. The moving particles generate an interference pattern which is advected by the flow ("Methods" section). This interference pattern was recorded with a camera mounted on a simple optical microscope in brightfield configuration^32^.

**Figure 1:**
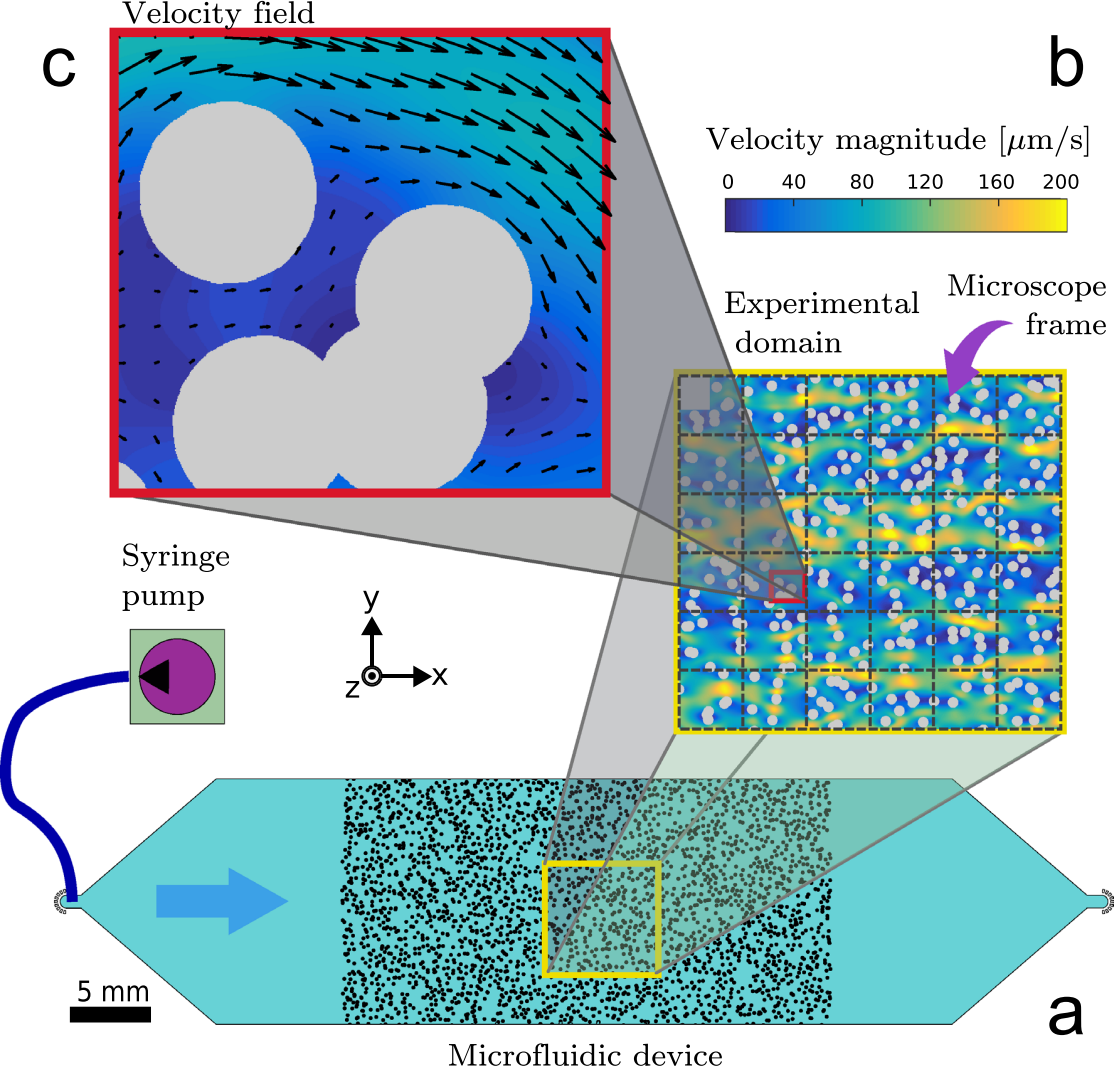
Experimental setup and velocity measurement. (**a**) Microfluidic device with a porous zone of $30~\text {mm }\times \,15~\text {mm}$ and depth of 100 µm containing pillars of 200 µm diameter. The experimental domain (flow field shown in (**b**)) has a size of $7~\text {mm }\times \text { 7 mm}$ and is marked with a yellow frame in (**a**). In order to achieve a very high resolution, the experimental domain was scanned and stitched together over 36 positions (gray dashed lines). (**c**) shows a close-up of the measured velocity field in a small portion of the experimental domain, illustrating the high resolution of our analysis where the vectors have been placed in every 40 th pixel of the gridded velocity field. The velocity fields shown correspond to a non-Newtonian flow with a flow rate of $q_{\text{in}}$ = 5 µL/min.

**Figure 2:**
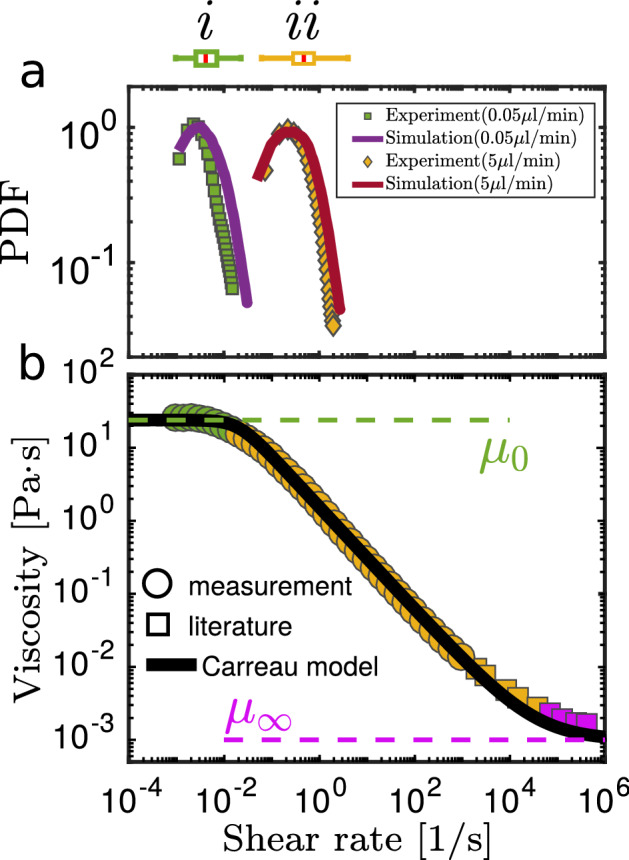
Measured shear rates and constitutive Law. (**a**) Shear rate distribution in our microfluidic device for $q_{\text{in}}$ = 0.05 µL/min (*i*) and $q_{{\text{in}}}$ = 5 µL/min (ii) with the corresponding box plots above. (**b**) Rheology curve of a 0.5 wt% xanthan solution. Our direct measurements (circles) were extended with literature values^40,41^ (squares) for extremely high shear rates. The black solid line corresponds to a Carreau model fit to the data with parameters $\mu _\infty =0.001\text { Pa}\, \text {s}$, $\mu _0 = 24\text { Pa}\, \text {s}$, $\lambda = 40\text { s}$ and $n = 0.3$. Green, yellow and violet symbols indicate the three regimes of the Carreau curve, where in the tails (green, violet) the apparent viscosity is almost constant at $\mu _0$ or $\mu _\infty$.

Our experiment consists of a microfluidic device containing a quasi-2*D* porous structure composed of randomly placed pillars, which are allowed to overlap creating a “swiss-cheese” type of pore structure^36^ with a packing fraction of 0.8 (Fig. 1a). As non-Newtonian fluid we use a 0.5 wt% xanthan gum solution. Xanthan is a polysaccharide mainly used in the food industry^37^ and for enhanced oil recovery^12^. In solution xanthan gum is transparent, which is essential to perform tracer based velocimetry. Additionally, Xanthan gum shows low elastic effects in shear driven flows at a given concentration^38,39^. Figure 1b shows the full velocity field measured in a section of the mid-plane of the microfluidic channel. The flow field has been obtained by stitching together 36 individual measurements (Fig. 1b, dashed lines) in order to provide a representative characterization of the spatial pattern of the velocity in the porous structure (Fig. 1b). Figure 1c shows a close-up of the velocity vectors and the velocity contours around the obstacles. Thanks to the high resolution of our velocimetry measurements, we are able to create a smooth representation of the flow field. This is essential for computing the local shear rates numerically. The symmetry of the system in the vertical direction allows us to neglect the z-component of the strain tensor because the vertical velocity derivatives vanish in the mid-plane ($\text{d}(\cdot )/\text{d}z$ $\approx 0$), where the flow velocity is maximal. Thus, the shear rate (Eq. 5) in the mid-plane simplifies to

6 $$\begin{aligned} {\dot{\gamma }} \approx {\dot{\gamma }}_{xy}= \sqrt{2\left( \frac{\partial u}{\partial x}\right) ^2+\left( \frac{\partial u}{\partial y}+\frac{\partial v}{\partial x } \right) ^2+2\left( \frac{\partial v}{\partial y}\right) ^2}, \end{aligned}$$

where *u* and *v* are the velocity components in *x* and *y* direction, respectively (Fig. 1). Figure 2a shows the probability density function (PDF) of the shear rates for our xanthan gum solution at two different flow rates: = 0.05 µL/min (case i) and = 5 µL/min (case ii). The solid lines represent the corresponding distributions from the numerical simulations for comparison (solid lines).

### Measurement of the constitutive law

In order to map the local viscosity based on the shear rates obtained from the velocimetry measurement, we need to invert the fluid’s constitutive behavior locally. To determine the viscosity-stress relation of our xanthan gum solution we use a strain- and time-controlled rheometer (*MCR 502, Anton Paar, Austria*) with a double gap geometry (*DG26.7*). Xanthan gum solutions are shear-thinning. The constitutive relation measured (Fig. 2, circles) is well parametrized by a Carreau model^38,42^

7 $$\begin{aligned} \mu ({\dot{\gamma }})=\mu _\infty +(\mu _0-\mu _\infty ) (1+(\lambda {\dot{\gamma }})^2)^{\frac{n-1}{2}}, \end{aligned}$$

where $\mu _0$ is the viscosity at zero shear and $\mu _\infty$ the viscosity for ${\dot{\gamma }}\longrightarrow \infty$. The parameters $\lambda$ and *n* are used to describe the transition behavior. For the used xanthan gum solution we find the following parameters: $\mu _\infty =0.001\text { Pa}\, \text {s}$, $\mu _0 = 24\text { Pa}\, \text {s}$, $\lambda = 40\text { s}$, and $n = 0.3$, as shown in Fig. 2b (solid line). The constitutive relation described by Eq. (7) can be divided into three regimes. For low shear rates, the apparent viscosity is almost constant with a value of $\mu _0=\lim _{{\dot{\gamma }}\rightarrow 0}\mu ({\dot{\gamma }})$ (Fig. 2b, green). At intermediate shear rates, the viscosity decays with a power-law $\mu \sim {\dot{\gamma }}^{n-1}$ as shown by the yellow symbols in Fig. 2b. In the limit of high shear rates, the viscosity of the xanthan gum solution approaches the one of water $\mu _\infty =\lim _{{\dot{\gamma }}\rightarrow \infty }\mu ({\dot{\gamma }})$ (Fig. 2b, violet). For xanthan gum this intermediate regime between $\mu _0$ and $\mu _\infty$ covers a range of more than six orders of magnitude in shear rates. The boxplots on the top of Fig. 2a mark the median (red), the 25% to 75% quantiles (box) and the 1% and 99% quantiles (bars) of the shear rate PDFs of our two non-Newtonian experiments and show that they cover very different regimes on the rheology curve. For the experiment at low flow rate $q_{{\text{in}}}$ = 0.05 µL/min (case i) the shear rates are mainly distributed in the low shear regime of the xanthan gum rheology. Hence, the viscosity is almost constant at $\mu _0 = 24\text { Pa}\, \text {s}$ resulting in an almost Newtonian behavior. In contrast, the shear rates are approximately hundred times higher for $q_{{\text{in}}}$ = 5 µL/min (case ii), thus covering the intermediate regime of the constitutive relation where nonlinear rheological effects become relevant. Here, the local viscosity is distributed highly heterogeneously within the interstitial pore space and varies over a wide range of values.

**Figure 3:**
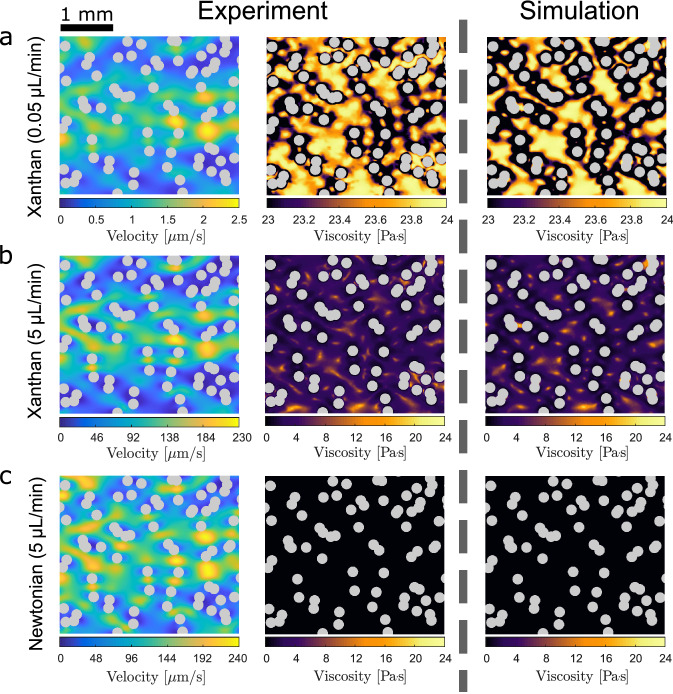
Velocity and viscosity maps at the pore-scale. The color range of the velocity fields (left) were scaled with the respective maximum value for better comparison. The middle column shows the viscosity fields in section of the mid-plane of our microfluidic device. (**a**) Xanthan gum solution at low flow rate (case i). (**b**) Xanthan gum solution at high flow rate (case ii). (**c**) Newtonian fluid (water) at a high flow for comparison. The mid-plane viscosities of the corresponding numerical simulations are plotted in the rightmost column for comparison and closely match the experimental results.

### Mapping viscosity at the pore scale

Combining the measurement of local shear rates with the measurements of the constitutive law of the xanthan gum solution allows us to map the local viscosity at the pore scale. Figure 3a,b show the measured velocity fields for the two cases i: $q_{{\text{in}}}$ = 0.05 µL/min and ii: $q_{{\text{in}}}$ = 5 µL/min, together with their corresponding viscosities (middle column). The local viscosity maps obtained from our numerical simulations ("Methods" section) are plotted in the right most column for comparison and closely matches the experimentally measured viscosity map. As shear rates increase with higher flow rates, different regimes of the constitutive relation shown in Fig. 2b are explored. Depending on the shear regime, small variations in the velocity pattern can considerably impact the shear and therefore the spatial distribution of the local viscosity. This effect is enhanced by the nonlinear viscosity shear relation, which enhances variations in shear in the intermediate regime while damping them in the low and extremely high shear case. At a flow rate of $q_{\text{in}}$ = 0.05 µL/min (Fig. 2, case i), shear rates are generally low and we barely observe variations in the local viscosity implying an almost Newtonian flow behavior. Only close to the obstacles boundaries the shear exceeds the threshold of $1/\lambda =10^{-2}~1/s$ causing the viscosity to decrease by about $1\text { Pa}\, \text {s}$ (Fig. 3a). For $q_{{\text{in}}}$ = 5 µL/min, the median shear rate is around ${\dot{\gamma }}\approx 0.8\gg 1/\lambda$, which lies well within the power-law regime of the xanthan gum rheology (Fig. 2, case ii). Consequently, the local viscosity varies over a wide range of values resulting in a highly heterogeneous viscosity pattern (Fig. 3b). Note the different ranges of the color scale in the two cases (a) and (b). Since the viscosity of xanthan gum approaches the viscosity of the solvent for very high shear rates ($\mu \approx \mu _\infty =\mu _{\text{water}}$), we also measure the velocity field of water alone at $q_{{\text{in}}}$ = 5 µL/min (Fig. 3c) to mimic the behavior of our solution in this limit. Reaching this limit in our experimental setup with the xanthan gum solution is impractical, as it would require flow rates which are far beyond the structural integrity of our microfluidic chip. Also the frame rates required to perform the velocimetry measurements would need to be five orders of magnitude higher than for the case $q_{{\text{in}}}$ = 5 µL/min. The velocity patterns show that the flow is more channelized in the non-Newtonian case at the high flow rate (Fig. 3b, left column), where also viscous resistance is more spatially variable. Note that, although the absolute values of the velocities shown in Fig. 3 are very different in the three cases, the color range was normalized to the interval $[0, v_{\text{max}}]$ to allow for comparison among the spatial patterns. Even tough the inlet flow rates are the same for the two cases b and c, the midplane velocity in the Newtonian case is usually higher. This behavior is caused by the vertical flow profile of the xanthan gum solution, which is flatter around the center than the parabolic profile of a Newtonian fluid^43,44^.

**Figure 4:**
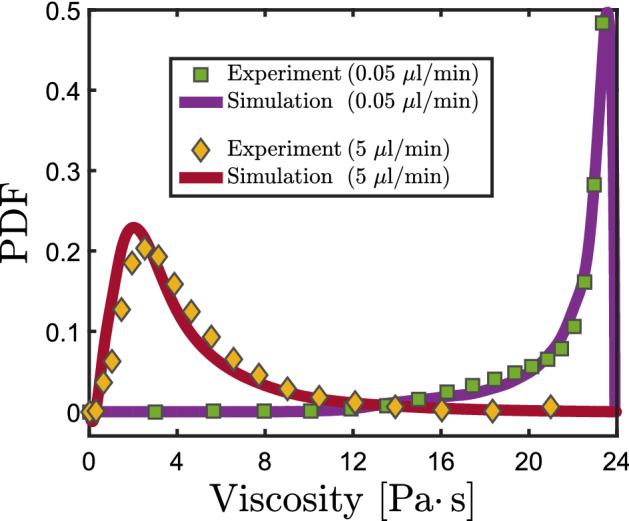
Probability density function (PDF) of the local viscosity in the mid-plane of the microfluidic chip. Green squares and yellow diamonds mark the two different experiments at flow rates $q_{{\text{in}}}$ = 0.05 µL/min and $q_{{\text{in}}}$ = 5 µL/min, respectively. In both cases the numerical simulations (solid lines) closely match the experimental behavior.

To get a quantitative comparison of the viscous variability shown in Fig. 3, we compute the probability density functions (PDF) of the measured local viscosity for the two non-Newtonian experiments and compare them with the viscosity statistics of the corresponding numerical simulations (Fig. 4). As expected from Fig. 3a the viscosity distribution clusters around the upper limit of $\mu _0= 24$ Pa s for the low flow rate ($q_{{\text{in}}}$ = 0.05 µL/min), while the viscosity distribution in case of the higher flow rate is considerably broader. Specifically, we find for the experiment a mean value of $\left\langle \mu ^{{\text{Exp}}} \right\rangle =23\text { Pa}\, \text {s}$ and a variance of $\sigma (\mu ^{{\text{Exp}}})=3.9$ for $q_{{\text{in}}}$ = 0.05 µL/min. A much lower mean of the viscosity ($\left\langle \mu ^{{\text{Exp}}} \right\rangle =3.8\text { Pa}\, \text {s}$) with an almost three times larger variance $\sigma (\mu ^{\text{Exp}})=10.1$ is obtained for the non-Newtonian experiment with $q_{{\text{in}}}$ = 0.05 µL/min. A similar behavior is also observed in the corresponding numerical simulations which closely follow the experimental results (Fig. 4). The fact that the experimental results compare well with the numerical solutions of the corresponding governing equations of motion shows that our method is able to detect even small variations in the local viscosity. Furthermore, it reveals that the constitutive law $\mu =\mu ({\dot{\gamma }})$ is generally suitable for our system and that adsorption, viscoelastic or viscoplastic as well as inertial effects can be neglected.

### Impact of the local viscosity on velocity distributions

**Figure 5:**
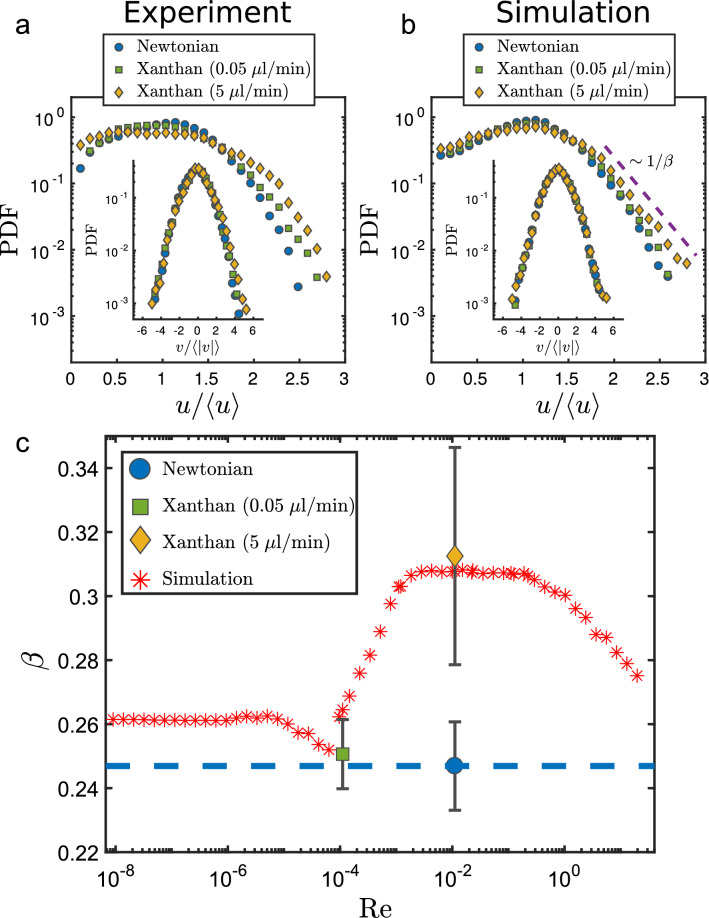
Impact of the local viscosity on the decay of flow velocity distributions. (**a**) Probability density function (PDF) of longitudinal velocity *u* and of the crosswise velocity *v* for the experimental results and (**b**) for the numerical simulation. The insets show the PDF of the crosswise component *v* of the corresponding cases. To compare the distributions at different flow rates, the velocities were normalized by their respective means. The longitudinal velocity distributions display exponentially decaying tails $P(u)\sim \exp (-1/\beta \cdot u/\left\langle u \right\rangle )$ where $1/\beta$ is the characteristic decay constant. (**c**) The parameter $\beta$ which describes the “heavyness” of the tail of the *x*-velocity distribution varies systematically as a function of Reynolds number. For low flow rates the xanthan gum solution behaves almost like a Newtonian fluid, thus displaying a decay which is independent of the flow rate. In the intermediate regime, where the viscous heterogeneity of the xanthan gum solution is high, the longitudinal velocity distribution is wider, corresponding to a larger $\beta$. Simulations at different flow rates are marked by red stars. Solid symbols indicate decay constants for the three experiments for low (green square) and high (yellow diamonds) flow rates of the xanthan gum solution and for the measured Newtonian case (blue). Error bars correspond to the 95% confidence interval of the linear fit of $1/\beta$ to the tail of the velocity PDF.

The broader viscosity distribution, measured for the non-Newtonian solution at the higher flow rate (Fig. 4, yellow diamonds), also affects the shape of the probability density function (PDF) of the longitudinal velocity. Consistent with previous observations, the *x*-velocity component *u* exhibits an exponential decay $P(u)\sim \exp (-1/\beta \cdot u/\left\langle u \right\rangle )$ in the case of Newtonian fluids^26,27^, both in the simulations and in the experiments (Fig. 5a,b). The discrepancies of the simulated and measured PDFs may root both in the necessary filtering/smoothing eminent of the PIV method and in the limited statistics for higher velocities. While the non-Newtonian cases also follow an exponential decay, the characteristic decay constant $1/\beta$, which is a measure for the channelization of the flow, changes systematically with the viscous heterogeneity in the flow. In the case of the low flow rate ($q_{{\text{in}}}$ = 0.05 µL/min), the non-Newtonian fluid behaves to a good approximation like a Newtonian one with constant viscosity equal to $\mu =\mu _0=24\text { Pa}\, \text {s}$. Consequently, the exponential decay of the normalized *x*-velocity distribution follows closely that of a Newtonian fluid. Note that, for a Newtonian fluid the shape of the (normalized) velocity distribution is independent of the applied flow rate as long as nonlinear viscous and inertial effects can be neglected (Stokes flow). This is confirmed by the shape of the velocity distribution measured for the low flow rate in which the non-Newtonian solution behaves like a Newtonian fluid even though the absolute flow rate of the Newtonian case is two orders of magnitude larger. For the high flow rate case ($q_{{\text{in}}}$ = 5 µL/min), where the viscosity of the non-Newtonian solution varies over several orders of magnitude (Fig. 3b), the *x*-velocity distribution is much broader. The heavier tail in this case is quantified by a larger exponential decay coefficient $\beta$ than for the other two cases (low flow and Newtonian). The broadening of the velocity PDFs is caused by the spatial variability of the local flow resistance which enhances preferential flow paths.

The characteristic decay constant $1/\beta$ of the probability density functions of the longitudinal velocities varies systematically with the Reynolds number ${\text {Re}}$ (Fig. 5c). Here the Reynolds number is defined as ${\text {Re}}=\rho ud/\mu _\infty$, where = 1,000 kg/m^3^ is the density of water, *u* is the mean flow velocity in the mid-plane at the entrance of the pore zone, $\mu _\infty =0.001\text { Pa}\, \text {s}$ is the viscosity at infinite shear, and ${d}=200$ µm is the diameter of the obstacles taken here as the characteristic length scale. In Fig. 5c, the red stars mark the results of extensive numerical simulations which span over a wide range of flow rates between and beyond the three experimental values which are plotted with green, orange and blue markers respectively. The trend of $\beta$ derived from the numerical simulations follows the rheological regimes of the xanthan gum solution shown in Fig. 2. The results are consistent with the experiments carried out under the corresponding flow conditions. At low Reynolds numbers, the viscosity is almost constant and barely affects flow properties. Consequently, $\beta$ is independent of $\text {Re}$ and constant at $\beta \approx 0.26$. Such a behavior is expected for Newtonian creep flows for which the normalized velocity distributions are invariant with $\text {Re}$. Increasing the flow rate results in higher shear rates ultimately surpassing the threshold of $1/\lambda$ beyond which nonlinear constitutive effects become relevant. Here the local viscosity varies over a wide range of scales. This increase of the heterogeneity in the viscosity comes with a broadening of the flow velocity PDFs, where $\beta$ increases by approximately 20% to $\beta \approx 0.31$. The broadening effect of the velocity PDFs is a fingerprint of increased channelization. At even higher flow rates, the viscosity approaches $\mu _\infty$, resulting again in narrower velocity distributions. The numerical simulations (red stars in Fig. 5) clearly show this systematic trend. Note that all our experiments and simulations are in the creep flow regime where inertial effects and turbulence can be neglected. We would also like to stress that the $\beta$ values computed from the experiments agree with the numerical results for both Newtonian and non-Newtonian fluids (Fig. 5c, filled symbols) within the measurement and calculation errors associated with the determination of $\beta$ ("Methods" section).

## Conclusion

The way how rheology controls flow properties in porous media has only been recognized recently, mainly through numerical simulations^21^, and, in most cases, using two-dimensional models^20,22^. Understanding how the fluid flow velocity varies as a consequence of the heterogeneity of the local viscosity in the interstitial pore space is of fundamental importance for many applications, since it fundamentally controls transport properties such as the channelization of flow paths or local diffusion coefficients of solutes. These rheological controls have important consequences for transport, mixing, and chemical reaction rates^3,15^ which cannot be infered from averaged bulk measurements.

Our experiments show that with high resolution velocimetry techniques it is possible to accurately measure the velocity, so that local shear rates in the flow of a non-Newtonian fluid through a complex structure can be duly computed. These measurements then allow us to map the local viscous resistance in the interstitial pore space, using the independently measured constitutive law of the fluid. This unique combination of two independent measurement techniques provides access to the local viscosity, which could not be experimentally measured in complex flows so far. The good agreement between experimental results and numerical simulations demonstrates the accuracy of our method. Moreover, we show that the exponential decay of the longitudinal velocity distribution, previously observed for Newtonian fluids^26,27^, varies systematically with the spatial heterogeneity of the local viscosity in the flow. By changing local flow velocities the distribution of local shear rates broadens and consequently, due to the non-Newtonian behavior, also the viscous heterogeneity increases. This variability of local viscous resistance as a function of the flow rate affects flow channelization and transport processes, as shown experimentally in the present work. This understanding may prove useful in many applications, such as the dimensioning and designing of filters, catalysts, and packed bed reactors, the determination of pollutant concentrations in groundwater remediation and the optimization of flow manipulation in enhanced oil recovery.

We anticipate that this combination of microfluidic experiments and rheology measurements can be used to quantify the influence of more complex rheologies, i.e., viscoelastic or viscoplastic, on the flow field in porous structures and provide experimental insight into the complex interplay between constitutive relations and hydrodynamics at the pore scale. While we restricted our microfluidic measurements to the horizontal components in the mid-plane of our device, the tools and methods we developed can be readily adapted to fully three-dimensional studies and to porous structures with porosities corresponding to real porous media^45^. The possibility of measuring the local viscosity with micrometric resolution opens new horizons in the study of the non-Newtonian fluid flow and in the analysis of viscous controls of transport properties in complex geometries.

## Methods

### Experimental methodology

The two-dimensional mask for the microfluidic chip was created by iteratively placing disks of unitary radius randomly on a rectangular domain measuring 300 × 150 radii. The disks are allowed to overlap. Every time a new disk is placed in the domain the overlap with previous disks is checked and the void space volume is recalculated using Monte-Carlo integration. This procedure is repeated iteratively until the desired porosity of $\phi = 0.8$ is reached. Using boolean operations, the disks were first unified and then subtracted from the domain. The technical drawing was rescaled to a size of 30 mm × 15 mm (corresponding to a pillar diameter of $d$ = 200 µm) and then used as a mask to produce a mold for the polydimethylsiloxane (PDMS) device. The microfluidic PDMS device was fabricated using standard soft lithography techniques^46^. The microchannel was etched to a depth 100 µm and then plasma-bonded to a glass slide. The chip is connected to a syringe pump (*Harvard Apparatus*) through which a fluid-tracer particle mixture can be injected (Fig. 1a). This setup allows to adjust the flow rate within a wide range of values and is only limited by the structural integrity of our microfluidic chip and the frame rate of the camera.

The non-Newtonian fluid used in our experiment is a 0.5 wt% xanthan gum solution. This solution is prepared by first dissolving xanthan powder in water. After degasing, one obtains a transparent solution allowing to perform image based velocimetry measurements. As tracers we use polystyrene particles (*Microparticles GmbH*) suspended in the xanthan gum solution. These particles have a diameter of $d_p=202 \pm 6$  nm and a density of 1.05 g/cm^3^, which matches the density of the xanthan gum solution to avoid sedimentation. Due to the small size of the tracer particles and their low concentration (10^−4^ wt%), the particles hardly affect the flow.

The experimental setup to record the tracer particle mixture is composed of a standard inverted bright-field microscope (*Ti-Eclipse, Nikon, Japan*) equipped with a CCD camera (*Hamamatsu ORCA-Flash 4.0 V3*). Ghost partlcies velocimetry requires a closed condenser of the microscope to observe the interference speckle pattern. The monochromatic light consequently increases the depth of field. Hence it is required to open the condenser as far as possible and still get enough signal of the moving speckle field. Buzzaccaro et al.^32^ showed, that GPV can be used to calculate accurate flow velocities for distinct focus planes in a flow profile. Images are acquired using a × 10-magnification with a resolution of 0.65 µm/pixel on a 2048 × 2048 pixel image and a depth of field of approximately 20 µm, and a frame rate of 100 fps. The use of the GPV technique allows us to obtain a final velocity field at a resolution of 5 µm in the observation plane. Consequently we have 40 datapoints per pillar diameter which is high enough to calculate accurate spatial derivatives.

**Figure 6:**
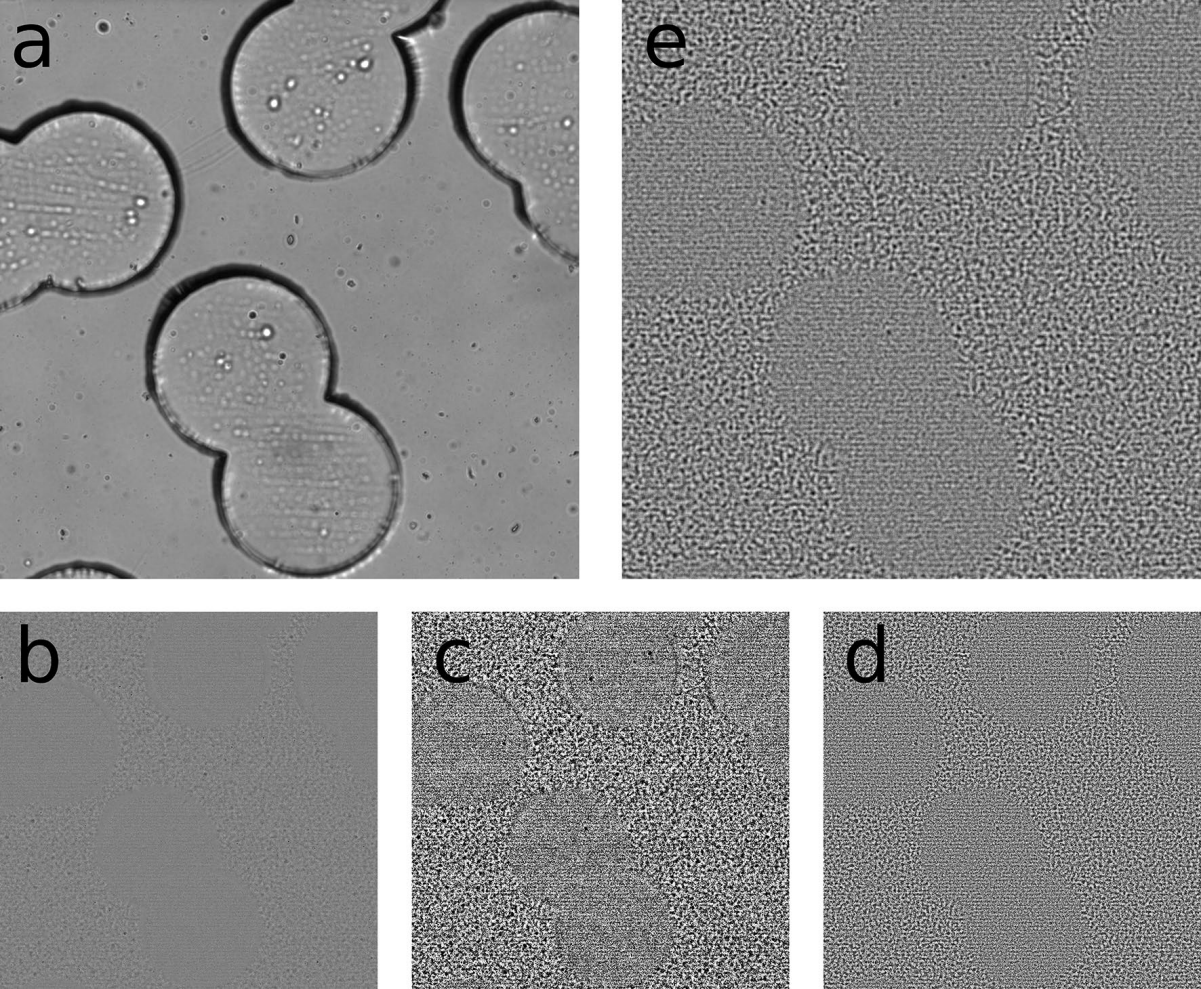
GPV images. (**a**) Image recorded during the experiment. (**b**) After subtracting the background from image (a) the speckle pattern in the interstitial pore space becomes clearly visible. (**c**–**e**) show the preprocessing of the images. (**c**) shows the CLAHE filtererd speckle pattern and (**d**) illustrates the high passed filtered image. Finally, (**e**) shows the final, Wiener filtered, image used for the PIV algorithm.

The images recorded during the experiment were analyzed using the $\text {Matlab}^{\text{TM}}$ toolbox *PIVLab*. First the normalized intensity of the whole image sequence is calculated and then subtracted from the single images to remove the background and obtain the moving speckle pattern (Fig. 6b). For pre-processing the images, we applied *PIVLab*’s CLAHE filter with a window size of 50 pixel (Fig. 6c), followed by a high-pass filter with window size of 15 pixel(Fig. 6d). Finally, a Wiener filter with window size of 6 pixel was applied to the image sequence (Fig. 6e). The CLAHE filter and the Wiener filter help to increase the signal of the decorraltion and the high-pass filter reduces the contribution of the speckles which are out of focus. The high pass-filter together with the fact that the in-focus signal is dominating over the off-focus contributions allows to accurately determine the velocity field of the mid-plane.

The cross-correlation analysis was also performed within *PIVLab*, using a Fourier transform with spline window deformation and 3 passes of 64 px, 32 px and 16 px. For more details on pre-processing filters and correlation algorithms we refer to the *PIVLab* user instructions^47^. For each of the 1500 images, we obtain an estimate of the velocity field for the whole panel, which are averaged to create the final data set. To meet the no-slip boundary condition on the obstacles, we co-registered the CAD mask with the GPV images and set the velocity values on the boundaries of the mask to zero before applying a low-pass filter. This filtering is required to de-noise the edges where the panels are stitched together and get a smooth representation of the flow field. The resulting data was then interpolated onto a final grid with 1 µm/pixel resolution using $\text {MATLAB}^{\text{TM}}$ 2D spline interpolation for further analysis. The interpolation to a grid with higher resolution is required to get a smoother representation for the spatial derivatives used for the shear rate. The velocity in stagnant zones is difficult to reconstruct because of the low number of particles exploring them. Here the cross correlation does not work properly and hence the velocity field may not be accurate. Due to the fact that these areas are difficult to identify analytically and are not relevant for our analysis, we simply accept the errors. The area of these stagnant zones is negligible compared with the area of the bulk, where we have highly accurate measurements. Hence, these errors do not influence our statistics. However, the velocity in these regions is very low and so is the resulting shear. Thus the viscosity should approach the lower limit of $\mu =\mu _0=24$ Pa s even if the velocity result is not very precise. From the PIV processing and the interpolation, we obtain a gridded representation of the *x* and *y* components of the velocity field with pixel size 1 µm microns. Based on these gridded velocity fields, we calculate the local shear rates using a second order finite-difference scheme. The characteristic decay coefficients $\beta$ were determined by making a linear fit to the tail of the longitudinal velocity PDFs on a semi logarithmic scale. The error reported in Fig. 5c denote the 95% confidence bounds of this linear regression.

### Computational simulations

All our computational simulations were performed by solving the three-dimensional (stationary) generalizations of the Navier–Stokes Eqs. (1)–(5) using a second-order finite-difference scheme as implemented by Ansys $\text {Fluent}^{TM}$^25^. Fluent allows to define non-Newtonian constitutive laws of the form $\mu ({\dot{\gamma }})$ parametrized by a Carreau model given by Eq. (7).

The numerical mesh was created by first importing the two-dimensional CAD model of the microfluidic mask into Ansys’ meshing module and creating a two-dimensional quadrilateral mesh. The mesh was then extruded vertically using 10 layers to obtain a three-dimensional discretization. The total computational mesh is composed of over ~ 30 million cells with an average cell volume of less than 2 × 10^−6^ mm^3^.

Finally the *x*, *y* and *z*-dimensions were scaled to match those of the microfluidic device. As boundary conditions for the flow, we used a velocity-inlet, a pressure-outlet and on all other walls non-slip boundary conditions. This mesh was then loaded into $\text {Fluent}^{TM}$, where we set the inlet velocity and the fluid properties using the fitted Carreau model of the rheology measurement (Fig. 2). As convergence criterion for the simulations, we used a residual threshold of 10^−6^.

Mid-plane values of the numerical simulation have been extracted by slicing the three-dimensional velocity field in the horizontal plane at half distance between the top and bottom of the microfluidic chip. Velocity and viscosity values reported for each computational mesh cell were directly exported and used as source data for the generation of Figs. 3 and 4 without the need to interpolate their values on an image grid.

## Data Availability

The datasets generated during and/or analysed during the current study are available from the corresponding author on reasonable request.
